# Cost-effectiveness of Simple Insulin Infusion Devices Compared to Multiple Daily Injections in Uncontrolled Type 2 Diabetics in the United States Based on a Simulation Model

**DOI:** 10.36469/9789

**Published:** 2018-08-22

**Authors:** Peter Wahlqvist, Jay Warner, Robert Morlock

**Affiliations:** 1 CeQur (Wales) Ltd, Life Science Hub Wales, Cardiff, Wales, United Kingdom; 2 CeQur Corporation, Marlborough, MA, USA; 3 YourCareChoice, White Lake, MI, USA

**Keywords:** cost-effectiveness, diabetes, device, insulin, infusion, type 2

## Abstract

**Background:**

As type 2 diabetes (T2D) progresses, administering basal and bolus insulin through multiple daily injections (MDI) is often required to achieve target control, although many people fail to achieve target levels. Continuous subcutaneous insulin infusion (CSII) treatment with traditional pumps has proven effective in this population, but use remains limited in T2D due to CSII cost and complexity. A new class of simple insulin infusion devices have been developed which are simpler to use and less expensive. This paper assesses at what price one such simple insulin infusion device, PAQ^®^ (Cequr SA, Switzerland), may be cost-effective compared to MDI in people with T2D not in glycemic control in the United States.

**Methods:**

Published equations were used in a simulation model to project long-term cost-effectiveness over 40 years, combined with data from the recent OpT2mise study, assuming similar efficacy of CSII and simple insulin infusion. Cost-effectiveness was pre-defined in relation to per capita gross domestic product (GDP), where incremental cost-effectiveness ratios below 1X the per capita GDP per quality-adjusted life year (QALY) gained were defined as “highly cost-effective” and below 3X GDP per capita as “cost-effective.”

**Results:**

Simple insulin infusion resulted in 0.17 QALYs gained per patient compared to MDI, along with lifetime cost-savings of USD 66 883 per person due to reduced insulin use and less complications. Analyses on price sensitivity of simple insulin infusion indicated that a device such as the PAQ is cost-effective compared with MDI up to price points of around USD 17 per day.

**Conclusions:**

For people with T2D not in glycemic control on MDI, simple insulin infusion devices such as PAQ have the potential to be highly cost-effective in the United States.

## Background

Type 2 diabetes (T2D) is characterized by a progressive decline in pancreatic beta-cell function and insulin secretion.^1^ As endogenous insulin secretion decreases, people with T2D require additional medications including insulin to maintain adequate glucose control, defined as hemoglobin A1c (HbA1c) < 7 by the American Diabetes Association.^2^ The progression of T2D is reflected in the treatment paradigm by which individuals progress through diet and exercise to one, two and frequently three anti-diabetic agents before beginning insulin therapy. Around 50% of people diagnosed with T2D require insulin therapy 6 years after diagnosis.^3^ Often insulin is added to the therapy of oral anti-diabetic drugs as a single daily injection. If HbA1c levels are not in target control, a basal-bolus regimen of insulin with multiple daily injections (MDI) of insulin is often required to achieve glycemic control.^4^

Although MDI has the potential to achieve target glycemic control, it is often challenging for people to adhere to this treatment, which includes injections outside of the home.^5^ Among people using insulin, 50% report skipping injections because injections interfere with their daily activities, result in injection pain and cause embarrassment.^5^ Healthcare providers are also challenged with intensifying insulin delivery as fewer than half of individuals that warrant insulin intensification (HbA1c >9%) are actually progressed.^6^ This can result in significant delays in intensification or absence of any intensification. A recent study showed a median time to first intensification of 17 months in a population with HbA1c 8.0% to 9.9% and 10 months in those with HbA1c >10%. Around 20% of the study population did not receive any treatment intensification at all.^7^ The result of these factors is that around 70% of adults with diabetes on insulin have difficulty achieving adequate glycemic control (AIC <7%).^8^ When glucose levels are sub-optimally controlled, people are at increased risk for diabetes-related complications that include damage to the eyes, kidneys, nerves, and cardiovascular system. Long-term randomized clinical trials have shown that early and persistent control of plasma glucose concentrations prevents and/or delays the development and progression of these complications.^9^

For people with T2D who are not able to attain glycemic control with MDI, continuous subcutaneous insulin infusion (CSII) with patient-controlled short-acting insulin boluses may be recommended.^10^ Compared to MDI, CSII has been shown to be safe and effective at improving glycemic control.^11^ Moreover, people using a CSII regimen report increased satisfaction and improved quality of life.^12^–^15^ A retrospective database study reported that CSII compared to MDI was associated with significant reductions in the use of anti-diabetic drugs, emergency room visits and inpatient admissions, resulting in improved care for individuals with T2D.^16^ Several studies concluded CSII should be considered for people with T2D who are not able to achieve glycemic control with MDI^11^,^17^,^18^ and a recent meta-analysis verified that CSII treatment results in better glycemic control as well as decreased use of insulin and no weight change.^19^

CSII can be delivered with traditional pumps, however these pumps can be complex and require lengthy training to use appropriately. They are not recommended for use in people with T2D in clinical guidelines from the American Diabetes Association^2^ and are in clinical practice mainly used in people with type 1 diabetes.^20^ Many of the functionalities in traditional pumps (i.e. multiple basal and bolus rate settings, bolus calculators) are not utilized by the T2D population and there is evidence that pumps with a limited number of fixed basal rates and simple bolus dosing will be acceptable for most people with T2D.^14^,^21^,^22^ Simple insulin infusion devices are not complex with intuitive and easy to use user interfaces.^19^ Simple insulin infusion devices deliver a pre-set amount of insulin into the body continuously and allow people to provide bolus doses with the push of a button.^23^ While there have been no randomized control studies, retrospective and uncontrolled studies report that simple insulin infusion demonstrated significant improvements in glycemic control, reduction of insulin dose and improved patient reported outcomes.^23^–^26^ Similar to traditional CSII pumps, the simple insulin infusion device may overcome the challenges associated with injectable insulin therapy. If simple insulin infusion devices provide a cost-effective treatment alternative compared to MDI, simple insulin infusion devices may become a recommended standard treatment alternative in selected populations with T2D.^10^,^19^

The current study was conducted to assess at which price a simple 3-day insulin infusion device called PAQ^®^ (CeQur SA, MA) may be cost-effective compared to MDI over a range of willingness to pay thresholds (i.e. cost per Quality-Adjusted Life Year (QALY) gained thresholds).

## Methods

### Data Sources

Equations and other data generated from the established United Kingdom Prospective Diabetes Study (UKPDS)^27^ were used in a model (software Microsoft Excel^©^) to project long-term complications, life-expectancy and quality-adjusted life years (QALYs) over 40 years for two treatment alternatives - using a simple insulin infusion device versus MDI. Clinical performance data used for the treatment alternatives were from the OpT2mise study,^11^ a randomized control trial conducted in 331 subjects with T2D. The study data were also used to represent simple insulin infusion compared with MDI, with regard to reductions in HbA1c and decreased insulin use. Baseline data on patient characteristics and comorbidities from the CSII arm in the OpT2mise study were used as input variables at start for both treatments in the UKPDS equations (Table 1).

Briefly, the UKPDS equations^27^ use data for age, sex, ethnicity, duration of diabetes, height, weight, smoking status, total cholesterol, high density lipoprotein (HDL) cholesterol, systolic blood pressure and HbA1c to predict estimates for the first occurrence of a complication. The complications estimated were myocardial infarction, ischemic heart disease, stroke, congestive heart failure, amputation, renal failure, blindness and mortality.

The Excel model was designed as an individual-based simulation.^28^ For each treatment alternative (simple insulin infusion and MDI), 10 000 iterations (representing a sample of 10 000 individuals) were made. These iterations were then repeated 20 times to account for the variance from each run and to ensure the stability of the model results. To avoid overestimating treatment effects on complications it was assumed that there is a linear decrease in differences between treatments until they converge (i.e. become zero) after 40 years. Utility values were obtained from a study^29^ that in a later review^30^ was identified to best represent standards set by the National Institute of Clinical Excellence (NICE).

### Cost Data

The cost of insulin for each treatment alternative was calculated using the daily doses from the OpT2mise study,^11^ and insulin prices were determined from Medispan PriceRX.^31^ In the MDI arm, all costs (August 2017 US dollars [USD]) included insulin, needles and insulin pens. Device costs were not included in the simple insulin infusion arm and were computed later to assess price sensitivity. Results from the OpT2mise study^11^ and the associated estimations of annual insulin costs are shown in Table 2. Baseline HbA1c was 9.0%. After the 6-month study period, AIC levels in the CSII group were 7.9%, which was significantly lower than the MDI (8.6%) group. The CSII group also had a significantly lower total daily insulin dose (97 IU/day) compared to the MDI group (122 IU/day). Based on these doses, the annual insulin costs (August 2017) were estimated to be USD 9757 for the simple insulin infusion group and USD 14 086 for the MDI group (including needles and pens).

The costs of complications in the US (Table 3) were obtained from a systematic literature review^32^ and inflated from 2015 to 2017 (August) using the US Consumer Price Index. “Cost of incident event” from the systematic review (Table 2 in Zhu *et al*. 2016)^32^ was used for the 1st year. The mean annual incremental costs for years 1 to 5 following the event calculated and used for subsequent years. The cost for angina was used as a proxy for ischemic heart disease. A general annual cost of USD 2000 for managing a diabetes patient was assumed and included, in order not to overestimate benefits of a longer survival. Costs and outcomes were discounted by 3% per year in order to compare the costs and outcomes that emerge over 40 years at today’s present value.

### Cost-Effectiveness

Cost-effectiveness was pre-defined in relation to per capita gross domestic product (GDP) in accordance with the WHO CHOICE framework.^33^,^34^ Incremental Cost Effectiveness Ratios (ICERs) below 1x GDP per capita and 3x GDP per capita per QALY gained were defined as ‘highly cost-effective’ and ‘cost-effective’, respectively. In the US, the GDP per capita was obtained from the World Bank for 2016 (USD 57 467)^35^ and used as the threshold.

ICERs were expressed as incremental cost per QALY gained in USD according to:

$$\text{ICER}=({\text{COST}}_{\text{Simple insulin infusion}}-{\text{COST}}_{\text{MDI}})/({\text{QALY}}_{\text{Simple insulin infusion}}-{\text{QALY}}_{\text{MDI}})$$

Where COST is the lifetime cost of either simple insulin infusion or MDI and QALY represents simulated quality-adjusted life expectancy with simple insulin infusion or MDI respectively. The price of the simple insulin infusion device was then set at levels which resulted in costs per QALY gained corresponding to the values 1x GDP per capita, 2x GDP per capita and 3x GDP per capita. This way, the price sensitivity of the device could be investigated over a range of cost per QALY gained thresholds.

The robustness of the estimated ICERs were tested in a series of sensitivity analyses including varying discount rates between 0% and 6%, reducing simulation time to 20 and 30 years, reducing and expanding efficacy of CSII as observed in the OpT2mise study by 50%, reducing and expanding the effect on insulin dose observed in the OpT2mise study by 50% as well as reducing and increasing complication costs by 50%.

## Results

### Estimates of Survival Rates, Event Rates and Quality-Adjusted Life Years

Over the 40-year time-period, simulations showed that simple insulin infusion compared to MDI was associated with a longer life expectancy of 0.32 years (Table 4). In addition, the simple insulin infusion group had 0.37 years longer event-free survival. The quality adjusted life expectancy was estimated to be 0.30 years longer for the simple insulin infusion group compared to the MDI group.

The simulation predicts individuals in the simple insulin infusion group to have considerable reductions in lifetime risk of developing diabetes-related complications compared to the MDI group (Table 4). For example, estimations indicate a 23.7% relative reduction for amputation and 11.3% relative reduction for blindness in the simple insulin infusion group compared to the MDI group. The overall relative risk reduction for any CV event was 8.7%. The all-cause mortality over 40 years was similar between the two groups, and 99.8% of people in both arms died within the 40-year time horizon, thus ensuring that the results were representative of life time outcomes and costs in the both simulated groups.

### Estimated Cost of T2D Using Simple Insulin Infusion and MDI Regimens

Cost estimates for managing T2D with simple insulin infusion or MDI are shown in Table 4 based on the simulations. The estimates show a USD 65 335 reduction in drug costs as well as a reduction in the cost of complications, resulting in lifetime discounted savings of USD 66 883.

In order to estimate at what daily price simple insulin infusion would be cost effective, the QALYs and estimated total costs (Table 4) for the simple insulin infusion and MDI groups were used to compute the threshold. The GDP per capita for the US was USD 57 467 in 2016. Applying the definition of cost-effectiveness chosen,^33^,^34^ a simple insulin infusion device would be cost effective (3x GDP per capita) at a daily cost per patient of USD 16.8, at USD 15.1 when using 2x GDP per capita and highly cost-effective (1x GDP per capita) at the maximum daily cost per patient of USD 13.4.

### Sensitivity Analyses

**Figure 1:**
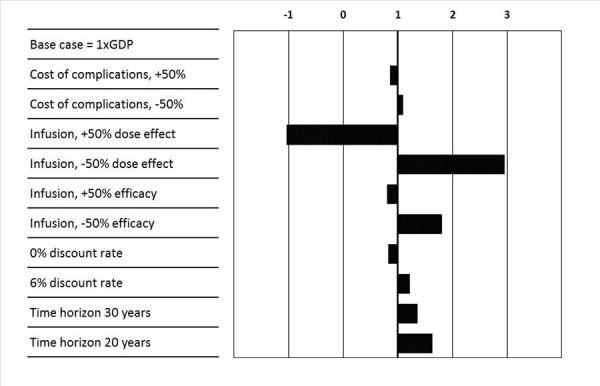
Sensitivity Analysis Presented as ICERs Representing Fractions of GDP per Capita, Where Results Below 1x GDP per Capita Are Highly Cost-effective and Below 3x GDP per Capita Are Cost-effective at a Daily Cost of a Simple Insulin Infusion Device of USD 13.4 per Patient GDP: gross domestic product; ICERs: incremental cost effectiveness ratios; USD: United States dollars

The parameters varied in the sensitivity analyses are shown in Table 5. ICERs were estimated using the USD 13.4 daily cost of simple insulin infusion therapy and defined in deviations from GDP per capita per QALY gained (Figure 1). The estimated ICERs were within the cost-effectiveness threshold of 3x GDP per capita in all sensitivity analyses. The estimates were most sensitive to varying the treatment effect on insulin doses. Expanding the efficacy of simple insulin infusion had a lower effect on cost-effectiveness than reducing the efficacy. Lowering the time horizon to 30 years impacted cost-effectiveness marginally, as most differences on life expectancy would be realized within this time horizon and as a result of discounting costs and effects. Lowering the time horizon to 20 years had further effects on cost-effectiveness, as the effects on QALYs gained in this time frame were not fully realized.

## Discussion

This study was conducted to compare the cost-effectiveness of a simple insulin infusion device to MDI for individuals with T2D not in glycemic control on MDI, where effectiveness data were obtained from the OpT2mise trial.^11^ The results indicate a simple insulin infusion device will be highly cost-effective in the United States at a price of around $13 per day and remain cost-effective up to a price of around $17 for people with T2D.

We only considered direct medical costs for the healthcare provider in this study, and the results would support greater cost-effectiveness if other types of costs were included. Examples of other costs include indirect costs of premature mortality, costs for sick leave, presenteeism costs, transportation costs, and nursing/home care costs.^36^ Further, costs of informal care (e.g. by spouses or other relatives) are usually not considered in diabetes studies, even if these may impose a substantial burden for the individual informal caregiver.^37^

Unit costs for diabetes complications such as CV events, amputations, blindness and renal failure vary substantially depending on the cost source. This is a well-known phenomenon^38^ and may be due to several factors, such as study methods used to assess costs or the payer perspective. We used a recent study that reviewed unit costs irrespective of assessment methods or payer perspective. Sensitivity analyses were made that showed a minimal effect on results of the level of unit costs used for diabetes complications in the current study.

The design of this study on the cost-effectiveness of simple insulin infusion and the assessment of a device price requires that we use a cost-effectiveness threshold. In this case, we have used the GDP per capita as a basis to determine what the acceptable threshold is, below which a treatment alternative can be considered cost-effective. The WHO has suggested that a treatment that averts one disability-adjusted life-year for the cost of 1x GDP per capita in a country or region is considered to be “very cost-effective,” while it at a cost of 3x GDP per capita is still considered “cost-effective”.^33^ These values have since then frequently been used in studies as thresholds to determine cost-effectiveness. Although this interpretation has rightfully been criticized as the sole ground to assess cost-effectiveness of new treatments in different countries,^34^ it does provide a benchmark that can be applicable in the US. For about 20 years, a cost-effectiveness threshold of USD 50 000 per QALY gained has been used and considered a lower limit. In recent years, a threshold up to around 3x GDP per capita has been suggested to be an upper limit.^39^ Hence, we provided results for the US using the range of thresholds between 1x GDP per capita and 3x GDP per capita.

We used clinical data from the relatively recent OpT2mise study,^11^ since it is the largest randomized clinical trial made of CSII vs MDI in T2D. Another important factor is that a dose-optimization run-in period was made in the OpT2mise trial to reduce bias before randomization. The clinical results used in this study agree in general with previous reports using a disposable insulin delivery device^24^,^25^,^26^ to show these devices are associated with improved glycemic control. Cost-effectiveness results from this study are also in agreement with another study on the cost-effectiveness of CSII vs MDI in the Netherlands,^17^ which used the IMS CORE Diabetes Model (CDM) and data from the OpT2mise trial.^11^ The CORE model study showed larger simulated effects on life expectancy (0.54 years vs 0.18 years in this study), QALYs gained (0.43 years vs 0.16 years in this study) as well as higher direct cost offsets due to fewer complications in the CSII arm of EUR −11 081 (USD −14 600, 2013) compared to USD −1975 in this study. This suggests that the estimates from the current model are very conservative compared with potential estimates made using the CORE model. For example, if a QALY gain of 0.43 years is used instead of 0.17, the simple insulin infusion device price per patient and day that is cost-effective would be estimated to around USD 25 instead of USD 17. Results when comparing this model to the CORE model are supported by a recent systematic review of the relationship between improved glucose control and modelled health outcomes.^40^ Results in terms of increased QALYs and life expectancy per percent decrease in HbA1c were higher when using the CORE model compared with results using other models. Nonetheless, a multivariate regression analysis including all models predicted a 0.37 increase in QALYs per percent decrease in HbA1c, which would correspond to a 0.26 QALY difference between treatments in the current study.

The sensitivity analyses showed results to be most sensitive to the reduction in insulin doses that can be obtained by using simple insulin infusion. Again, we argue that the current analysis is conservative. For example, if the difference in QALYs between treatments is 0.43 like the study by Rose *et al*^17^ suggests, this would have resulted in an ICER of 1.08x GDP per capita instead of the current 2.95x GDP per capita in the sensitivity analysis on dose reduction. That is, simple insulin infusion would in that case still be highly cost-effective also when the effect on dose reduction is halved.

A limitation with this study is the assumption that management of T2D will remain relatively stable over time. It is possible that diabetes treatment and management may change as a result of advances in medical technology or increased life expectancy. There is probably even more uncertainty around what will be the future prices of insulin. Another limitation is the fact that this modeling study was based on a clinical trial, which may not account enough for real-life factors such as compliance. That this is a modeling study is a limitation by itself, since it may not adequately mirror costs and outcomes in clinical practice.

Further, it is not certain exactly to what extent the UKPDS study is applicable for a US population. The UKPDS study was -when initiated- the largest trial designed to allow the creation of independent risk equations for diabetes type 2 complications, and the UKPDS study is still used as a landmark study for the validation of results from more recent studies.^41^ The American Diabetes Association has also used statements referring to “the UKPDS Outcomes Model,” which is based on the UKPDS study equations.^42^ Moreover, a US study found predicted all-cause mortality based on UKPDS data comparable with that of observed US data.^43^

The most important limitation is the assumption that the difference between treatment alternatives in insulin doses and HbA1c levels will be maintained over time. However, a 1-year follow-up of the OpT2mise study indicated that differences between treatments in terms of HbA1c and dose reductions remained stable over time.^44^

## Conclusions

For people with T2D not in glycemic control on MDI, a simple insulin infusion device has the potential to be highly cost-effective in the United States, which would enable these devices to become a valuable treatment alternative in this population.

## Figures and Tables

**Table 1:** Population Characteristics at Baseline in the Group that Received CSII Treatment in the OpT2mise Study^11^

Baseline Data	CSII (n=168)
Age, years	55.5 (9.7)†
Years living with diabetes	14.9 (8.0)
Hemoglobin A_1c_ (%)	9.0 (0.8)
Systolic blood pressure, mm Hg	132.3 (15.2)
Diastolic blood pressure, mm Hg	75.6 (9.4)
Total cholesterol, mmol/L	4.5 (1.4)
HDL cholesterol, mmol/L	1.2 (0.4)
LDL cholesterol, mmol/L	2.2 (0.8)
Triglycerides, mmol/L	2.3 (2.4)
Body Mass Index (kg/m^2^)	33.5 (7.5)
Females, %	44
Blacks, %	4
Smokers, %	14
Dyslipidemia, %	16
Retinopathy, %	4
Hypertension, cerebrovascular disease, coronary heart diseases, %	85
Peripheral vascular disease, %	7
Diabetic nephropathy, %	13
Peripheral neuropathy, %	0

CSII: continuous subcutaneous insulin infusion

† Values are mean (standard deviation), unless otherwise noted.

**Table 2:** Hemoglobin A1c at Baseline and End of the Opt2mise Study (6 Months), Daily Insulin Dose at End of the Opt2mise Study,^11^ along with Estimated Annual Therapy Costs for Insulin, Needles and Pens

	CSII	MDI
Hemoglobin A1c at baseline (%)	9.0	9.0
Hemoglobin A1c at end of study (%)	7.9§	8.6
Total dose of insulin at end of study (units/day)	97§	122
Estimated annual therapy costs	$97 757†	$14 086

CSII: continuous subcutaneous insulin infusion; MDI: multiple daily injections

§ p<0.0001 compared with MDI

† Includes only cost of insulin as the device costs were to be determined by the analysis

**Table 3:** Cost of Complications from T2D Used in the Model Inflated to August 2017

Events	2015, 1st year, USD	2015, subsequent years, USD	2017, 1st year, USD	2017, subsequent years, USD
Ischemic heart disease	8159	4288	8452	4442
Myocardial infarction	30 181	4617	31 264	4783
Congestive heart failure	12 958	5530	13 423	5728
Stroke	13 682	3519	14 173	3645
Amputation	23 825	4659	24 680	4826
Blindness	2913	2913	3017	3017
End-stage renal disease	220 187	220 187	228 085	228 085

T2D: type 2 diabetes; USD: United States dollars

**Table 4:** Estimated Survival, Quality Adjusted Life Years, Estimated Event Rates at 40 Years and Estimated Costs (USD) of T2D Drugs, Management and Complications for People with T2D Using Simple Insulin Infusion and MDI

Survival and QALYs	Simple insulin infusion	MDI	Difference
Overall survival (years)	21.80	21.48	0.32
Event-free survival (years)	21.39	21.02	0.37
Discounted survival (years)	15.68	15.50	0.18
Undiscounted QALYs	16.42	16.12	0.30
Discounted QALYs	11.86	11.69	0.17
**Event rate at 40 years, %**	**Simple insulin infusion**	**MDI**	**Relative difference**
Ischemic heart disease	8.9%	9.5%	−5.9%
Myocardial infarction	17.7%	19.6%	−10.0%
Congestive heart failure	7.4%	8.2%	−9.7%
Stroke	5.3%	5.7%	−7.1%
Any CV event*	39.3%	43.0%	−8.7%
Amputation	6.0%	7.8%	−23.7%
Blind	4.0%	4.5%	−11.3%
Renal Failure	4.4%	4.4%	−0.1%
All-cause mortality	99.8%	99.8%	0.0%
**Costs**	**Simple insulin infusion**	**MDI**	**Difference**
Diabetes drug costs	153 030	218 366	−65 335
Diabetes management	30 819	30 373	428
Ischemic Heart Disease	7265	7367	−103
Acute Myocardial Infarction	6054	6471	−417
Congestive Heart Failure	2514	2719	−205
Stroke	1834	1888	−54
Amputation	2185	2728	−543
Blindness	957	1040	−83
Renal Failure	36 075	36 645	−570
Total	240 715	307 597	−66 883

USD: United States dollars; T2D: type 2 diabetes; MDI: multiple daily injections; QALY: quality adjusted life years; CV: cardiovascular

* The sum of ischemic heart disease, myocardial infarction, congestive heart failure and stroke.

**Table 5:** Parameters Varied in the Sensitivity Analyses

Sensitivity analysis	Parameter varied	Original value	Sensitivity value
Cost of complications, +50%	Complication costs	-	x 1.5
Cost of complications, −50%	Complication costs	-	x 0.5
CSII, +50% dose effect	Daily insulin dose	97 ml	84.5 ml
CSII, −50% dose effect	Daily insulin dose	97 ml	109.5 ml
CSII, +50% efficacy	HbA1c effect	−1.10%	−1.45%
CSII, −50% efficacy	HbA1c effect	−1.10%	−0.75%
0% discount rate	Discount rate	3%	0%
6% discount rate	Discount rate	3%	6%
Time horizon 20 years	Time horizon	40 years	20 Years
Time horizon 30 years	Time horizon	40 years	30 years

CSII: continuous subcutaneous insulin infusion
